# Polypoid nodular histiocytic hyperplasia associated with endometrioid adenocarcinoma of the endometrium: report of a case

**DOI:** 10.1186/1746-1596-9-93

**Published:** 2014-05-12

**Authors:** Shabnam Akhter, W Dwayne Lawrence, M Ruhul Quddus

**Affiliations:** 1 Department of Pathology and Laboratory Medicine, Women & Infants Hospital, Alpert Medical School of Brown University, 101 Dudley Street, Providence, Rhode Island 02905, USA

**Keywords:** Endometrium, Nodular histiocytic hyperplasia, Endometrioid carcinoma

## Abstract

A 45 year old woman underwent Laparoscopy-assisted total hysterectomy with staging procedure following a diagnosis of endometrial endometrioid adenocarcinoma on her endometrial biopsy. The hysterectomy specimen showed a FIGO I stage 1a, endometrioid carcinoma. *A separate polypoid lesion in the endometrium, distinct from the carcinoma, was also identified. Microscopically the polypoid lesion was “nodular histiocytic hyperplasia”.* The H&E, immunohistochemical staining findings and the differential diagnoses are discussed in this report. Although *description of* similar lesions is available in the literature, the current lesion is unique as it is identified in a hysterectomy specimen in its entirety and its association with an endometrial endometrioid carcinoma.

**Virtual Slides:**

The virtual slide(s) for this article can be found here: http://www.diagnosticpathology.diagnomx.eu/vs/1060511915121922

## Introduction

Nodular histiocytic hyperplasia, initially thought to be of mesothelial origin because of their sites of occurrences, was reported in hernia sac [1]. Subsequently similar lesions have been reported from various other body sites, e.g., the pericardial sacs and cardiac valves [2], peritoneum [3] and pleura [4]. Their presence in endometrial biopsy may mimic neoplasia. A recent series published seven cases of nodular histiocytic hyperplasia [5]. The cases, so far reported in the literature, are associated with benign lesions only. Since the previously reported cases were encountered in endometrial biopsies the lesions were fragmented. *We report here a case of a nodular histiocytic hyperplasia associated with FIGO I/III endometrial endometrioid adenocarcinoma. We also describe the lesion in its entirety; as noted above, all previous descriptions of this entity were based on their presence in biopsy specimens*.

### Case presentation

A 45 year-old woman with a history of uterine fibroids presented with vaginal bleeding. She was Gravida 1 and Para 0, with a history of termination of pregnancy. A pelvic ultrasound showed a 9.1 x 8.0 x 4.0 cm partially distorted uterus with multiple hypoechoeic nodular areas, the largest being 4 x 3 cm and a thickened, 1.8 cm, endometrial stripe.

### Pathologic findings

An endometrial biopsy revealed a FIGO I endometrial endometrioid type adenocarcinoma. After weighing-in all the options presented to her, the patient opted for a Laparoscopy-assisted vaginal hysterectomy with bilateral salpingo-oophorectomy and staging procedure.

**Figure 1:**
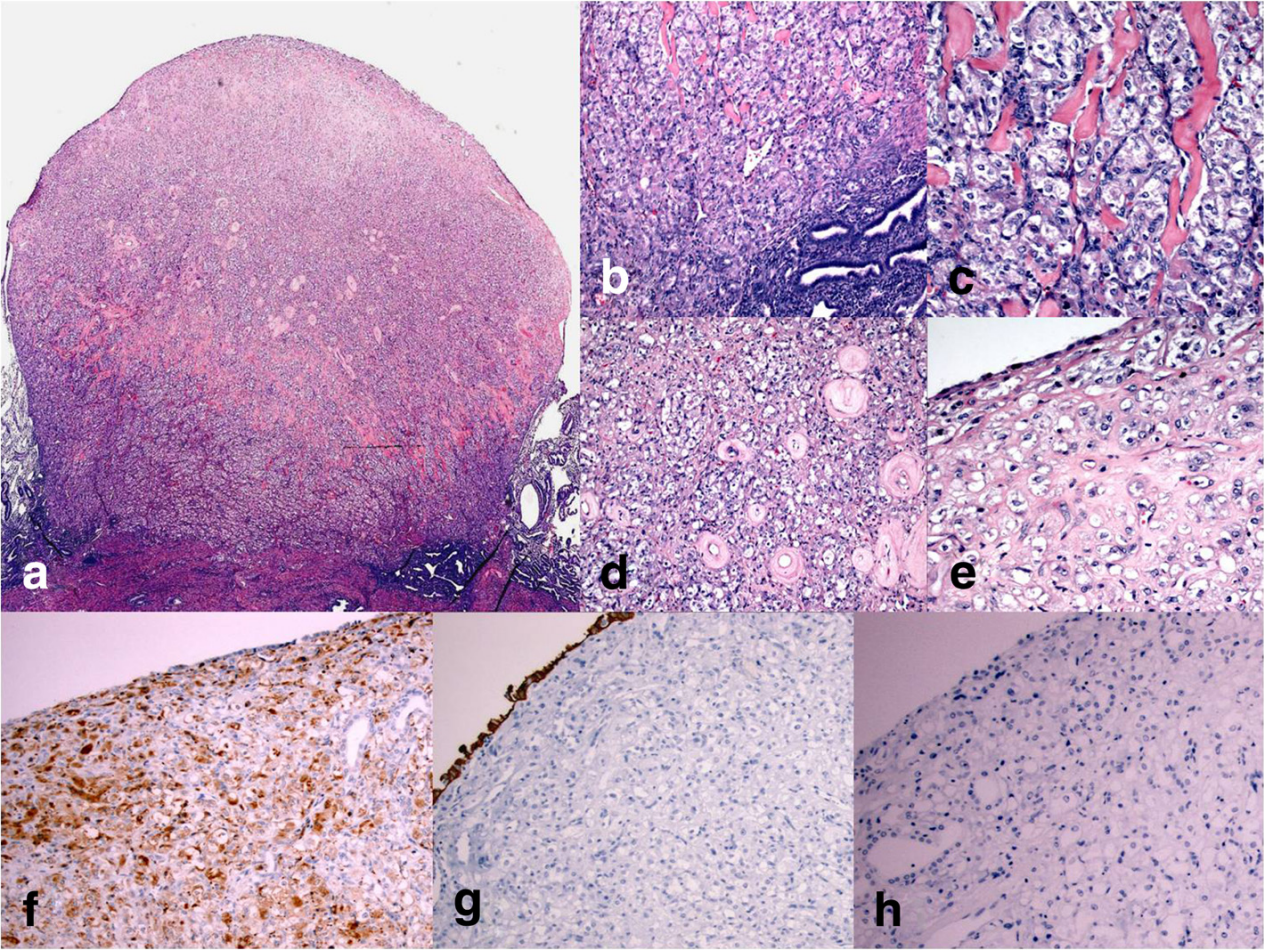
**Photomicrographs of Nodular Histiocytic Hyperplasia. **1**a** (H&E): Low power magnification of polypoid nodular histiocytic hyperplasia; 1**b** (H&E): Closely packed histiocytes near the base of the polyp; 1**c** (H&E): pink hyalinized fibrous bands separating aggregates of histiocytes; 1**d** (H&E): thick-walled blood vessel mimicking “onion-skinning”; 1**e** (H&E): more sclerosis towards the surface of the polyp; 1**f** (IHC): histiocytic immunohistochemistry marker (CD68) showing diffuse positivity; 1**g** (IHC): Pancytokeratin marker (AE1/AE3) is completely negative (surface epithelium is reactive serving as positive internal control); 1**h** (IHC): HMB45 is negative.

Gross examination of the specimen revealed a 172 gms, 9.6 x 6.5 x 5.6 cm distorted uterus with multiple subserosal and intramural fibroids. Upon opening the uterus, a 4.5 x 4.2 cm shaggy polypoid lesion was identified in the endometrial cavity involving both uterine walls. In addition a 0.7 x 0.5 cm polypoid lesion (Figure 1a) was found on the anterior wall of the endomyometrium with smooth surface. The fibroids were noted in the intramural and subserosal locations.

Microscopically, a smooth surfaced polypoid lesion was found to consist mostly of aggregates of histiocytes that were more closely packed near the endomyometrial junction, i.e., the base of the lesion (Figure 1b). Pink hyalinized fibrous bands are noted throughout the polypoid lesion (Figure 1c). Many blood vessels with thickened hyalinized walls are present throughout the lesion, more commonly towards the surface (Figure 1d). Towards the surface, the histiocytes were relatively sparse and separated by sclerosis (Figure 1e). The polyp was lined by a thin, attenuated, layer of endometrial epithelium (Figure 1e). The histiocytes resembled perivascular epithelioid cells. And presence of thick walled blood vessels within the lesion raised the question of a perivascular epithelioid cell tumor (PEComa). Immunohistochemical stains reveal that the lesional cells are strongly and diffusely reactive to a histiocytic marker, CD68, (Figure 1f) and non-reactive to HMB45 (Figure 1h), pancytokeratin (AE1/AE3), (1 g) Epithelial membrane antigen (EMA), Desmin, Smooth Muscle Actin (SMA), CD10, Vimentin.

**Figure 2:**
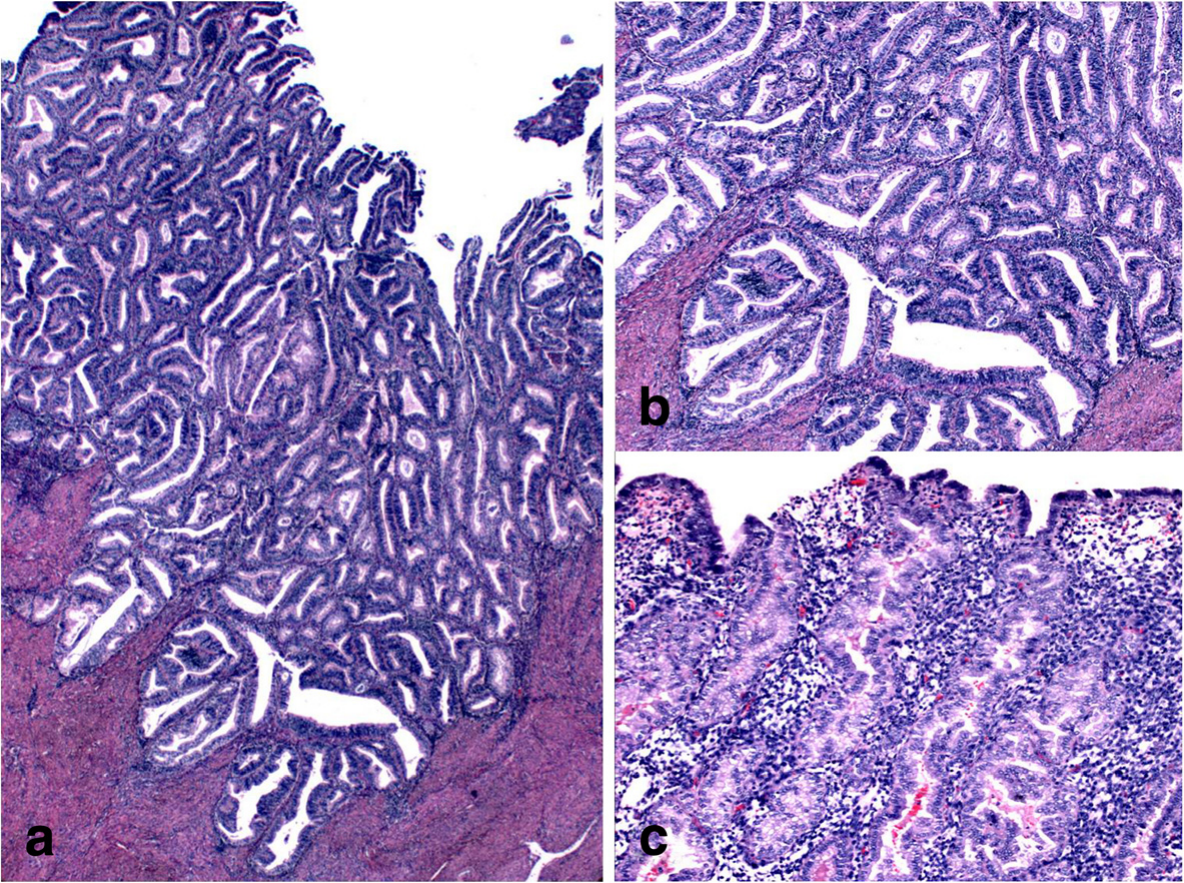
**Photomicrographs of Endometrioid adenocarcinoma arising in a background of secretory Endometrium.** 2**a** (H&E): Low power view of FIGO I endometrioid carcinoma; 2**b** (H&E): high power view of the same tumor; 2**c** (H&E): Carcinoma is arising in a background of secretory endometrium.

FIGO grade I stage 1a endometrioid adenocarcinoma was noted in the endometrium as well (Figure 2a and Figure 2b). The tumor involves areas of adenomyosis; however, true myometrial invasion was not present. The carcinoma was arising in background of functional endometrium (Figure 2c). The lower uterine segment and cervix were free of tumor. No lymph-vascular space invasion was identified. The bilateral ovaries and fallopian tubes were unremarkable. The regional lymph node dissection was all negative for metastatic carcinoma.

## Discussion and conclusions

Nodular histiocytic hyperplasia is a rare lesion *often incidentally encountered in endometrial biopsies. Majority of these lesions are* reported in the literature as single case report. The largest series, so far has been reported in the literature, is based on 7 cases [5]. The lesion, when encountered in endometrial biopsies and present in small fragments, may mimic a neoplasm. So far all the reported cases are, *however,* associated with benign diseases. *The current report is based on a case* of nodular histiocytic hyperplasia associated with a FIGO I/III endometrial endometrioid adenocarcinoma. The lesion itself is identified grossly in its entirety as a *distinct entity presenting as an endometrial polyp*.

The differential diagnoses of this entity include Langerhans cell histiocytosis, xanthogranulomatous endometritis, malakoplakia, signet-ring cell changes of the endometrial stromal cells, etc. Available reports in the literature have elaborated how to distinguish nodular histiocytic hyperplasia from their mimics. The current case showed some resemblance with perivascular epithelioid cell tumor (PEComa) or an epithelioid smooth muscle tumor with many small hyalinized blood vessels. *Microscopic and multifocal PEComa of the female genital tract have been reported *[6,7]*.* One interesting finding noted in the current case is the presence of hyalinized fibrous strands throughout the lesion; *a feature often seen in endometrial stromal tumors. The cytologic appearance of the tumor cells seen in endometrial stromal tumor is, however, completely different from what was seen in nodular histiocytic hyperplasia. The cells in endometrial stromal tumor are small, mostly round and darkly stained, especially in low grade stromal sarcoma*.

Special stains that were performed to rule out the mimics show the lesional cells of nodular histiocytic hyperplasia are strongly and diffusely reactive to histiocytic marker, CD68. All other markers including, epithelial markers (AE1/AE3), smooth muscle markers, and endometrial stromal cell marker (CD10) were non-reactive. HMB45, a marker for PEComa, was also negative.

*The site of nodular histiocytic hyperplasia was clearly located on the surface of endometrium with smooth lining and protruding into the endometrial cavity*.

It is speculated that the nodular histiocytic aggregates may result from previous endometrial biopsy. The current case did have a recent history of endometrial biopsy one month prior to her hysterectomy. The previous biopsy in this case revealed endometrial carcinoma with squamous differentiation and no evidence of histiocytic aggregate. It is also of note that the appearance of the foamy histiocytes often seen in endometrial biopsies is also different from the histiocytes present in nodular hyperplasia as they lack the foamy cytoplasm.

Mazur and Kurman [8] proposed that these histiocytes apparently reside in the endometrial cavity and reported their presence in association with hydrometra and benign bleeding patterns. The authors postulated that it may represent a response to what they have proposed as “intracavitary debris.” The current case was associated with an endometrioid adenocarcinoma so presence of some “intracavitary debris” is not unlikely. As noted before the polypoid lesion was seen projecting into the endometrial cavity.

A possibly related, but morphologically dissimilar, histiocytic endometrial lesion has been reported by Iezzoni and Mills in their study of non-neoplastic endometrial signet-ring cells [9]. No signet-ring cells are identified in this case.

Kim et al. proposed that the endometrial stromal cells showing progestational changes with atrophic endometrial glands trapped in the middle may produce histologic similarities that may vaguely resemble histiocytic aggregate [10]. *Unlike nodular histiocytic aggregate, decidualized stromal cells do not form a discrete nodule. Kim et al. also speculated that the nodules may not originate in the endometrium because no vasculature was seen in their cases *[10]*. The current case documents many blood vessels in the nodule and thus contradicts that speculation of Kim et al.*

In conclusion, nodular histiocytic hyperplasia may not always be associated with benign/inflammatory lesions as previously reported in the literature. The current case documents its association with endometrioid carcinoma of the endometrium.

The patient is currently followed routinely and is disease free 80 months after surgery.

### Consent

The patient has given consent for the use of the images and case presentation for educational and scientific purposes provided the unique patient identification is not revealed.

## Competing interest

The authors declare no competing financial interest. All the authors have actively participated in the diagnosis and manuscript writing.

## Authors’ contributions

SA is the Stuart Lauchlan International Visiting Fellow in Gynecologic and Breast Pathology and participated in writing up the case report and MRQ is the attending Pathologist on the case. WDL offered his expert opinion in finalizing the case. All authors read and approved the manuscript.
